# A simulation-based OSCE with case presentation and remote rating – development of a prototype

**DOI:** 10.3205/zma001594

**Published:** 2023-02-15

**Authors:** Lisa Bußenius, Sigrid Harendza

**Affiliations:** 1 Universitätsklinikum Hamburg-Eppendorf, III. Medizinische Klinik, Hamburg, Germany

**Keywords:** assessment, competence, OSCE, remote rating, simulation

## Abstract

Simulation-based examination formats improve the possibility to assess medical students’ competences during their performance. Additionally, videotaping of simulations allows for remote rating, providing advantages for raters, students, and exam organizers. We describe a simulation-based OSCE prototype with remote rating of students’ competences, developed to replace a conventional OSCE at Hamburg Medical Faculty. The assessment consists of two phases: a consultation phase with four simulated patient encounters and a case presentation phase where four students present two cases each. All encounters from the consultation and the presentation phase are to be videotaped and remotely rated by clinical raters. Advanced medical students (year 4) are to be assessed regarding their clinical knowledge as well as physician-patient-communication, clinical reasoning competence, and patient management competence. We provide detailed schedules for the simulation-based OSCE procedure and a roster for organization. When piloting the assessment, we encountered two major obstacles with respect to legal obligations regarding examination time and videotaping which allowed us to provide tips on how to successfully implement this assessment prototype. Remote rating will, when successfully implemented, help students to concentrate on their consultation or presentation tasks, reduce raters’ time constraints and also allow for randomized rating. Using established instruments for competence-rating rather than OSCE checklists provides an additional feature for this simulation-based OSCE prototype. Legal issues can be avoided by using the prototype for formative assessment but should be addressed in advance when it is planned to be used as summative assessment.

## Abstract (de)

Simulationsbasierte Prüfungsformate verbessern die Möglichkeit, Kompetenzen von Medizinstudierenden während ihrer Performanz bewerten zu können. Zusätzlich ermöglicht die Videoaufzeichnung von Simulationen eine Bewertung ohne Anwesenheit von Prüfenden (Fernbewertung), was Vorteile für Prüfende, Studierende und Prüfungsorganisierende bietet. Wir beschreiben den simulationsbasierten Prototyp einer OSCE inklusive Bewertung der Kompetenzen der Studierenden ohne Anwesenheit von Prüfenden, welcher entwickelt wurde, um eine konventionelle OSCE an der medizinischen Fakultät der Universität Hamburg zu ersetzen. Die Prüfung beinhaltet zwei Phasen: eine Sprechstundenphase mit vier simulierten Patient*innen und eine Fallpräsentationsphase, in der vier Studierende jeweils zwei Fälle vorstellen. Alle Gespräche aus der Sprechstunden- und Fallpräsentationsphase sollen auf Video aufgenommen und von klinischen Prüfenden nach Anschauen der Videos bewertet werden. Fortgeschrittene Medizinstudierende (8. Semester) sollen hinsichtlich ihres klinischen Wissens sowie ihrer Ärzt*innen-Patient*innen-Kommunikation, Clinical-Reasoning-Kompetenz und Patient*innenmanagement-Kompetenz bewertet werden. Wir stellen detaillierte Pläne für die simulationsbasierte OSCE und einen Ablaufplan für die Organisation zur Verfügung. Bei der Pilotierung des Projektes trafen wir auf zwei größere Hindernisse in Bezug auf rechtliche Einschränkungen hinsichtlich der Prüfungsdauer und der Videoaufzeichnung. Dadurch können wir Tipps für die erfolgreiche Implementierung dieses Prüfungsprototyps geben. Wenn erfolgreich umgesetzt, wird eine Abwesenheit der Prüfenden und spätere Bewertung der Videos den Studierenden dabei helfen, sich auf ihr Anamnesegespräch oder die Fallpräsentation zu konzentrieren, die zeitlichen Einschränkungen von Prüfenden reduzieren und außerdem eine randomisierte Bewertung ermöglichen. Etablierte Instrumente für die Kompetenzbewertung statt OSCE-Checklisten zu verwenden ist ein weiteres Merkmal dieses simulationsbasierten OSCE-Prototyps. Rechtliche Fragen können vermieden werden, wenn man den Prototypen als formative Prüfung einsetzt, sollten aber im Vorfeld geklärt werden, wenn die OSCE als summative Prüfung geplant wird.

## 1. Background

With the National Competence Based Catalogue of Learning Objectives for Undergraduate Medical Education [1], medical curricula in Germany become increasingly competence-based. This subsequently requires the development of competence-based assessment formats. Currently, objective structured clinical examinations (OSCEs) – checklist-based assessments in the presence of raters – are used to assess medical students’ skills [2]. To assess competences, simulation-based assessments with a more holistic rating approach can include greater task complexity in more authentic settings to allow expression of performance [3]. Additionally, videotaped simulations allow for remote rating to reduce raters’ influences on students’ performance [4]. Time-independent rating also allows for flexible work scheduling, which can reduce OSCE-related logistical issues and costs [5]. To assess advanced medical students’ competences in a summative exam, we developed a prototypical simulation-based OSCE including remote rating options. 

## 2. Objectives

Our main goal was to replace a traditional OSCE by a simulation-based OSCE to allow assessment of students’ competences. To design an OSCE prototype for this purpose we used an evidence-based approach [6]. Our target group were advanced medical students (year 4), who had acquired knowledge, skills, and attitudes needed to perform in a simulated setting. 

## 3. Developing the prototype

For our project, we chose an OSCE at the end of a fourth year learning module in the integrated curriculum at the University of Hamburg [7]. At the end of this module, students must be able to communicate with patients and take detailed histories, discuss differential diagnoses based on diagnostic results, and suggest therapeutic approaches. The designated module covers medical topics around the abdomen and retroperitoneum from the clinical disciplines gastroenterology, general surgery, nephrology, and urology. The final OSCE with about 200 students takes place over two days and includes seven five-minute stations and checklists for assessment of clinical knowledge and communication skills. 

**Figure 1:**
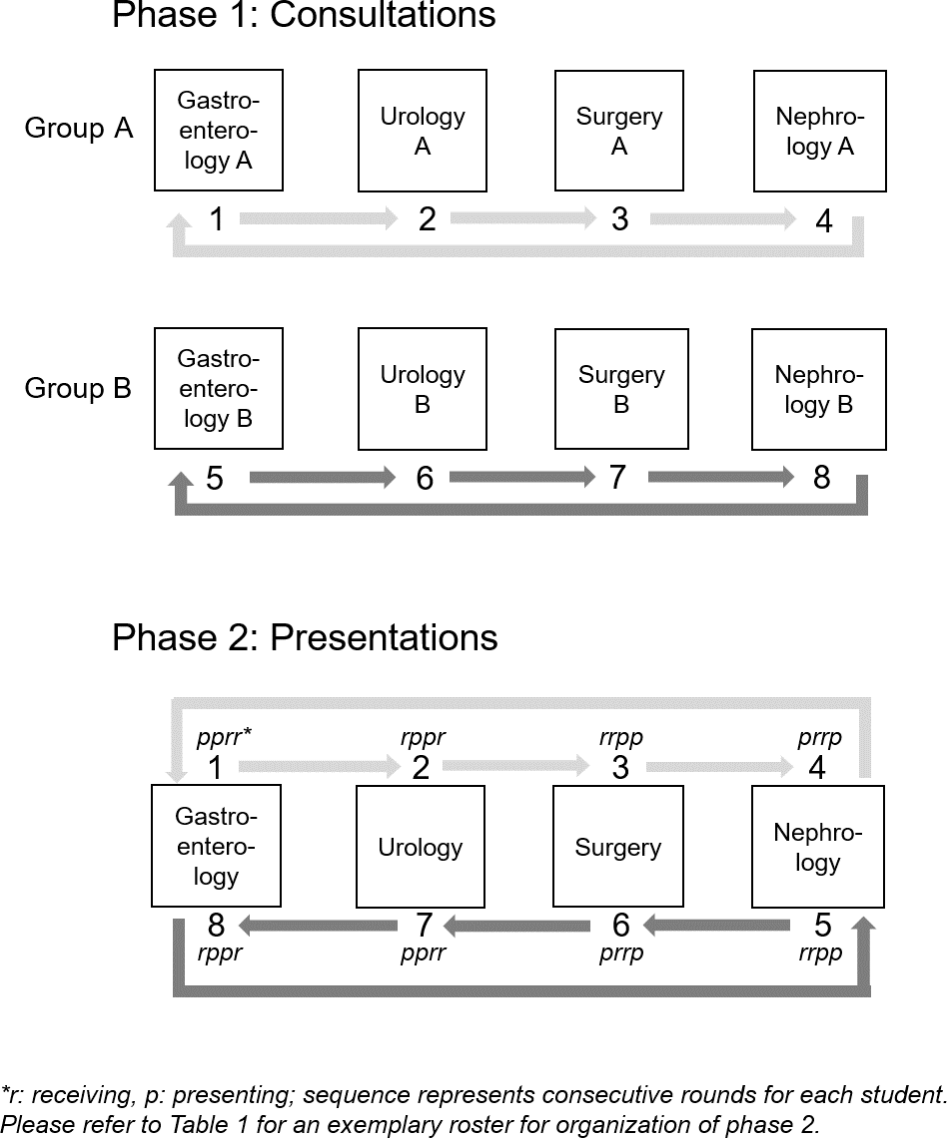
Rotation plans for phase 1 and 2 of the simulation-based OSCE

The aim of the OSCE redesign was to measure students’ clinical knowledge and communications skills by assessing their physician-patient-communication competence, clinical reasoning competence, and patient management competence while covering the clinical content of the module. For this purpose, we designed four stations simulating physician-patient encounters (phase 1: consultations) and four stations simulating patient-case presentations in handover situations (phase 2: presentations) based on similar steps used in a validated competence-based simulation of a first day in hospital [6] (see figure 1 (Fig. 1)). In phase 1 (consultations), eight students rotate through four 6-minute stations in two parallel courses, A and B. The students, who are randomly assigned to their starting positions, encounter different cases from each of the four clinical disciplines in the two courses. Students’ assignments for each station include taking focussed histories from simulated patients and discussing test results with them, which are presented during the patient interview. All encounters are videotaped which allows for remote assessment. Besides the respective medical content correctness, clinical reasoning and physician-patient communication competences can be remotely assessed with specific questionnaires [8], [9]. In phase 2 (presentation), groups A and B are combined, so that two students meet in one room for a 6-minute handover. Group B rotates in the opposite direction of group A to ensure an even mix of student pairs. Every student will present (p) and receive (r) two patients so that all four disciplines are covered in this phase (e.g., in round 1, student 1 presents (p) a gastroenterology case to student 8 who receives (r) it, while in round 2 student 8 presents (p) a nephrology case to student 3 who receives (r) it). Students’ assignment includes presentation and discussion of each case to come to a management conclusion. Analogous to phase 1, all handovers will be videotaped in phase 2 which allows for remote assessment. Besides assessing students’ knowledge of medical content, the presenting and the receiving student are also assessed with regard to their communication competence [9], [10], clinical reasoning [8], and patient management [11].

**Figure 2:**
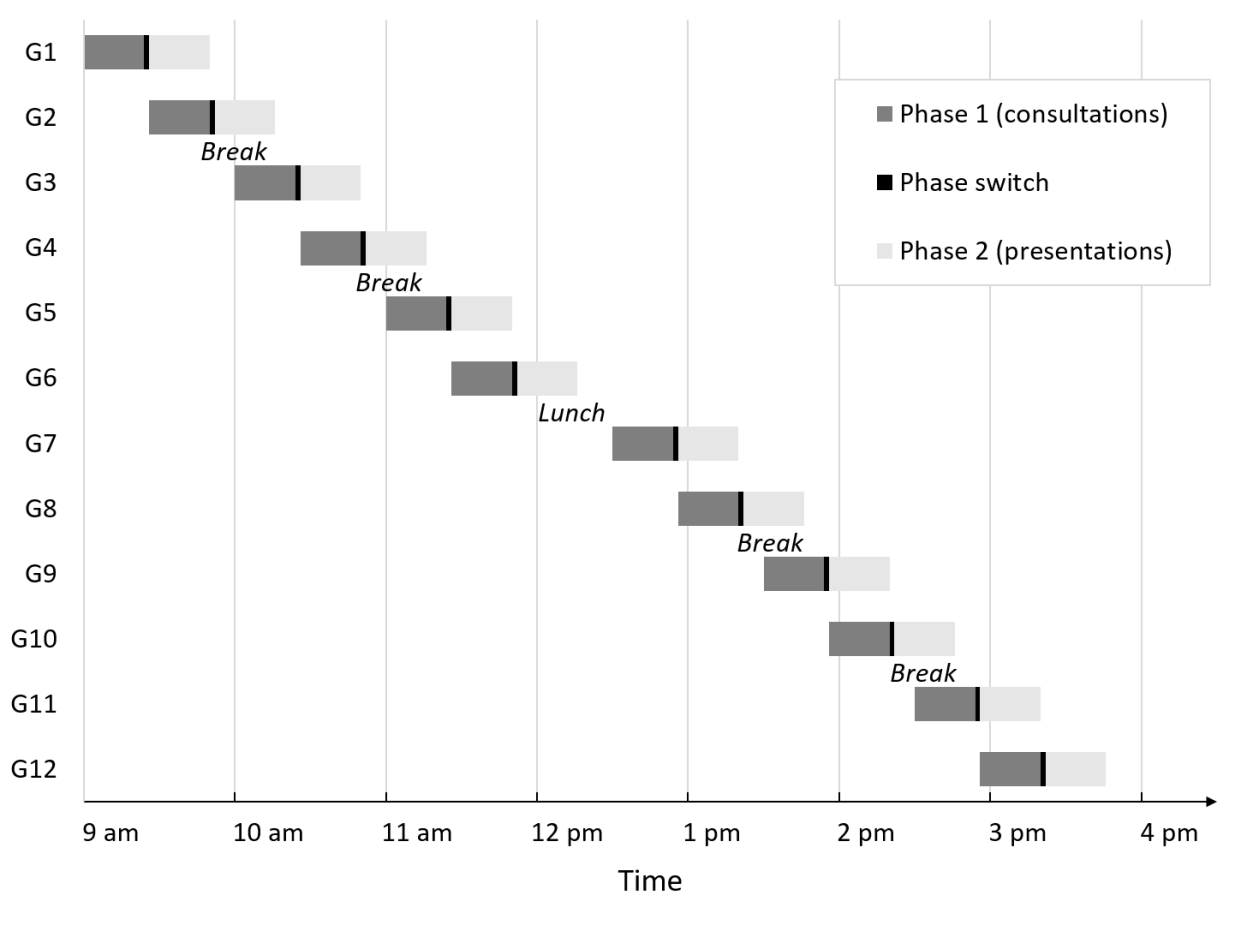
Schedule for one examination day of the simulation-based OSCE

With one minute for changing between the physician-patient encounters and between each handover and two minutes to change from phase 1 (consultation) to phase 2 (presentation), the simulation-based OSCE lasts 56 minutes. With overlapping phases (i.e. when group 1 enters the presentation phase, a new group can start with the consultation phase), 100 students can be examined in one day during normal working hours with reasonable breaks for the simulated patients (see figure 2 (Fig. 2)). With remote rating, no clinical raters need to be present and can perform the rating when they are off clinical duty. Every student will receive eight different ratings, four from phase 1 and four from phase 2, i.e. two from each discipline (see table 1 (Tab. 1)). 

## 4. Pitfalls with and tips for implementation

We piloted the simulation-based OSCE as summative assessment in July 2019 with 186 students [12]. However, due to time constraints in the exam regulations of our medical school, we were only allowed 42 minutes to incorporate seven subjects into our assessment. Thus, we were not able to use our planned design with the two phases, which would have needed 56 minutes. Tip 1: if you wish to use this prototype with a consultation and a presentation phase including changing times, check with the exam regulations of your medical school whether there are limitations for exam time. In our case, we had to stick to the traditional OSCE format and cancel the presentation phase.

Furthermore, we were not allowed to film the conversations which the students held with the simulated patients for legal reasons at our medical school. Therefore, the remote rating, a main feature of assessment in our prototype, could not take place. Tip 2: if you wish to use remote rating, check with your legal department whether it is possible to take videos of students for remote assessment. Audio tapes could be an alternative, if they are allowed.

However, at least we were able to keep the core idea of our project by introducing elements of simulation into the traditional OSCE format. Students entered the examination rooms not knowing which medical content of the four clinical disciplines to expect from a certain simulated patient, which requires good history taking skills. Tip 3: Adapt the patient cases from real cases and develop them together with experienced clinicians to increase content validity [13].

Instead of video-ratings, our examiners had to be present in the room for their ratings, but did not interfere in the student-patient-communication. Furthermore, due to the exam regulations at Hamburg Medical Faculty, which require a certain amount of points per OSCE station, the raters could not use the rating instruments for clinical reasoning [8] and physician-patient communication competences [9] we had planned for the consultation phase. However, instead of just rating medical content, we were at least able to integrate clinical reasoning and communication aspects in addition to medical content into our usual OSCE checklists. As an additional educational research project, raters with a background from psychosocial fields were permitted in the examination rooms to rate the students’ communication competence with the Global Rating Scale [9]. Tip 4: Before using the suggested instruments for communication competence, clinical reasoning, and patient management, check with your local exam regulations whether they will be allowed for summative assessment.

Due to legal regulations at Hamburg Medical Faculty, we were not able to perform the simulation-based OSCE with competence-based remote rating as a substitute for our usual summative OSCE. However, parts of the simulation, i.e. consultations and case presentations including remote rating of clinical reasoning, communication competence, and patient management have been demonstrated elsewhere to be successfully performed [8], [9], [10], [11]. The suggested prototype even works as telemedicine format which we implemented in an extended format as a formative assessment with feedback [13]. Time-independent, remote rating works well for the assessors’ work schedules. Furthermore, advantages of remote rating include that the videos can be assessed in a randomized order to prevent bias caused by order effects [14], and the assessors can use breaks at their own convenience to reduce rater fatigue [15]. Furthermore, blinding students’ to specific stations’ content helps to prepare them for real consultations where the cause of a patient’s problem is unknown. Additionally, remote rating requires less personnel on-site while the assessment takes place. When considering implementation of this OSCE format, we recommend to clarify anticipated barriers such as limited assessment time or possible legal issues with filming or data storage ahead of planning.

## Funding

This work was supported by the Federal Ministry of Education and Research (project number: 01GK1801A). The funding body played no role in the design of the study and in collection, analysis, and interpretation of data and in writing the manuscript.

## Ethical approval

The Ethics Committee of the Hamburg Chamber of Physicians confirmed the innocuousness of this study and its congruence with the Declaration of Helsinki (WF-047/16). The anonymity of all participants was guaranteed. 

## Acknowledgement

We thank all participating medical students, actors and actresses, raters, and the Deanery of the Medical Faculty Hamburg for their support of piloting this assessment format.

## Competing interests

The authors declare that they have no competing interests. 

## Figures and Tables

**Table 1:**
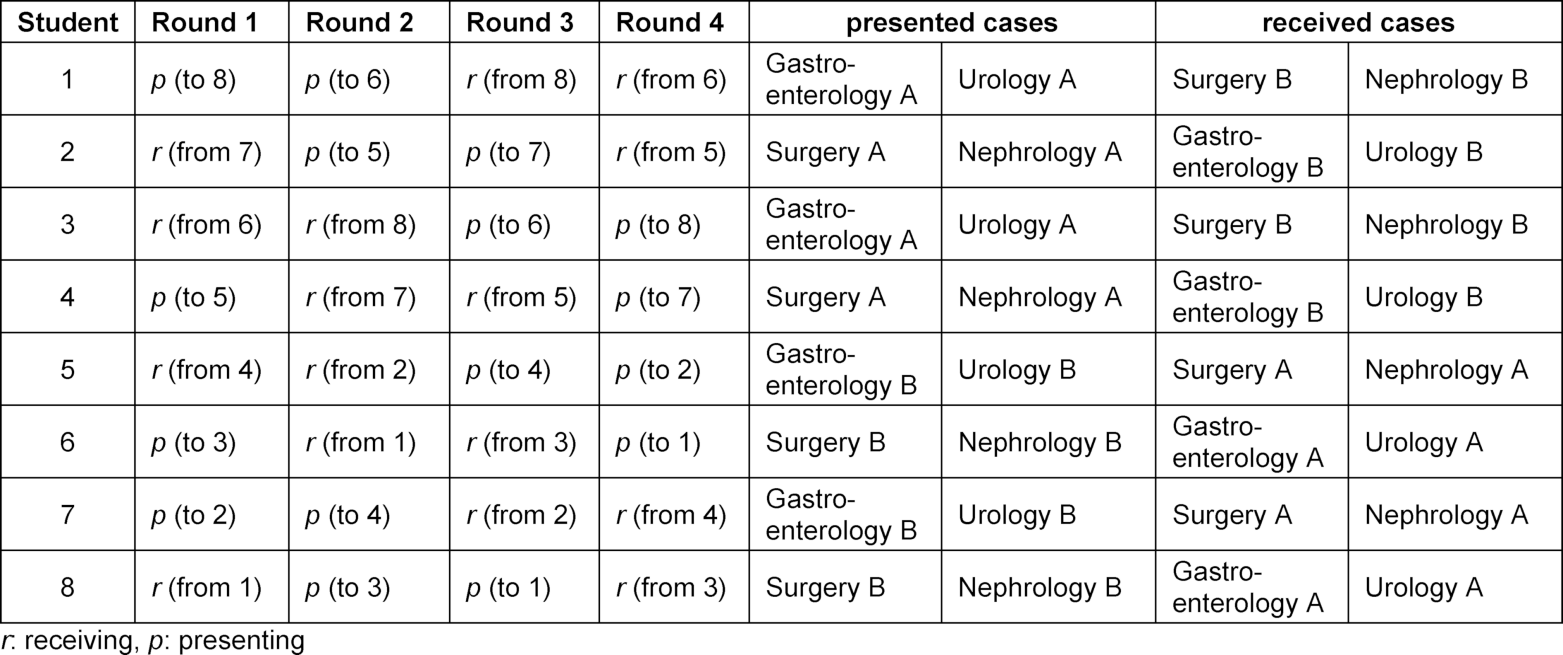
Exemplary roster for organization of phase 2 (presentations)
